# Prevalence of SARS-CoV-2 antibodies and associated factors in the adult population of Belgium: a general population cohort study between March 2021 and April 2022

**DOI:** 10.1186/s13690-024-01298-1

**Published:** 2024-05-15

**Authors:** Johan Van der Heyden, Victoria Leclercq, Els Duysburgh, Laura Cornelissen, Isabelle Desombere, Inge Roukaerts, Lydia Gisle

**Affiliations:** 1 Epidemiology and public health, Sciensano Juliette Wytsmanstraat 14, Sciensano, Brussels, 1050 Belgium; 2 Immune response, Sciensano, Brussels, Belgium; 3 Viral diseases, Sciensano, Brussels, Belgium

**Keywords:** Prevalence, Seroepidemiologic studies, SARS-CoV-2, COVID-19, Population, Belgium

## Abstract

**Background:**

This study assessed seroprevalence trends of SARS-CoV-2 antibodies in the Belgian adult population between March 2021 and April 2022, and explored factors associated with seropositivity and seroreversion among the vaccinated and unvaccinated population.

**Methods:**

A prospective longitudinal surveillance study was conducted within a random sample of the general population (18 + years) in Belgium, selected from the national register through a multistage sampling design. Participants provided a saliva sample and completed a survey questionnaire on three occasions: at baseline and in two follow-up waves. Outcome variables included (1) seropositivity, defined as the presence of SARS-CoV-2 antibodies, assessed with a semi-quantitative measure of anti-RBD (Receptor Binding Domain) IgG ELISA and (2) seroreversion, defined as passing from a positive to a negative antibody test between two measurements. Trends in SARS-CoV-2 antibody prevalence were assessed using binary logistic regression with contrasts applying post-stratification. Potential determinants of seropositivity were assessed through multilevel logistic regressions.

**Results:**

In total 6,178 valid observations were obtained from 2,768 individuals. SARS-CoV-2 antibody prevalence increased from 25.1% in the beginning of the study period to 92.3% at the end. Among the vaccinated population, factors significantly associated with higher seropositivity rates were being younger, having a bachelor diploma, living with others, having had a vaccine in the last 3 months and having received a nucleic-acid vaccine or a combination. Lower seropositivity rates were observed among vaccinated people with a neurological disease and transplant patients. Factors significantly associated with higher seropositivity rates among the unvaccinated population were having non-O blood type and being non-smoker. Among vaccinated people, the seroreversion rate was much lower (0.3%) in those who had received their latest vaccine in the last 3 months compared to those who had received their latest vaccine more than 3 months ago (2.7%) (OR 0.13; 95%CI 0.04–0.42).

**Conclusions:**

The rapid increase in antibody seropositivity in the general adult population in Belgium during the study period was driven by the vaccination campaign which ran at full speed during this period. Among vaccinated people, seropositivity varied in function of the time since last vaccine, the type of vaccine, sociodemographic features and health status.

**Supplementary Information:**

The online version contains supplementary material available at 10.1186/s13690-024-01298-1.

**Table:** 

**Text box 1. Contributions to the literature**	
• Further insights are provided on determinants of SARS-CoV-2 seropositivity at population level in both the vaccinated and unvaccinated population.	
• In the general population seroreversion after COVID-19 vaccination is extremely rare within the first three months after vaccination, but non negligible if the vaccination occurred more than three months ago.	
• Public health systems benefit by integrating seroprevalence studies in the general population in their surveillance systems.	

## Background

The first case of SARS-CoV-2 (severe acute respiratory syndrome coronavirus 2) infection in Belgium was reported on February 4th 2020 [1]. The rapid increase of people testing positive thereafter marked the first epidemic wave, which started on March 1st 2020 [2]. A national COVID-19 (Coronavirus Disease 2019) surveillance system was set up by Sciensano, the Belgian institute of health, at an early stage of the epidemic, mainly focusing on COVID-related cases, hospitalisations, deaths, and later, on vaccination coverage [2]. In parallel, several serological studies were launched according to the WHO recommendations [3]. Former research had indeed shown that specific serum antibodies to SARS-CoV-2 increased 2 to 3 weeks following the primary infection and remained detectable for 3 to 6 months after [4, 5]. Serology tests could thus be used to evaluate the number of people, including asymptomatic persons, that got infected with the virus and to estimate the cumulative prevalence of infection and disease transmission over time (6). Seroepidemiological studies could also provide an important empirical input for mathematical models in the analysis and prediction of the pandemic (7). During the vaccination campaign, serological surveillance remained relevant for assessing the prevalence of SARS-CoV-2 infection among the unvaccinated people and for comparing the immune response status between naïve and previously infected individuals among the vaccinated (8). The SARS-CoV-2 seroprevalence studies in Belgium were conducted in several settings, at first on residual blood samples (9) but also on specific subpopulation sera, e.g. from blood donors (2), healthcare workers in hospitals (10), primary healthcare providers (11), school children (12) and nursing home residents and staff [13, 14]. This paper reports findings of a seroprevalence study conducted in a general population sample of adults randomly selected from the Belgian national register. Similar serological studies in community settings were initiated in other European countries [15]. Ours took place in the period running from March 2021 to April 2022, with three data collection points within the same study sample.

Although serum-based methods are the gold standard to assess the presence of SARS-CoV-2 antibodies, research using blood samples is difficult to implement in a geographically scattered random selection of the general population. The major barriers encountered in this context are low participation acceptance as well as high economic, logistical and time constraints related to drawing blood samples and delivering them to a lab in optimal conditions. This led us to consider an alternative method for community-based surveillance, i.e., using salivary antibody tests. Two independent studies from the US and from Belgium had shown this to be a non-invasive, scalable substitute to serology tests [16, 17]. Another study confirmed that both serum and salivary IgG antibodies to SARS-CoV-2 persisted in the majority of COVID-19 patients for at least 3 months after symptom onset [18].

During the course of our study, Belgium faced four COVID-19 waves and a steep increase in the COVID-19 vaccination rate [2]. The study objectives were adapted to the rapidly progressing dynamics of the epidemic and considered the impact of the vaccination campaign on antibody prevalence. Three research questions are tackled in the framework of this paper:

- How did the prevalence of SARS-CoV-2 antibodies evolve during the 13-months study period in the general adult population, and independently among the vaccinated and unvaccinated people?
- What sociodemographic and health-related characteristics associate with seropositivity, in both the vaccinated and unvaccinated people?
- To what extent did people with a positive test result serorevert to a negative test result in a following data collection point, and what factors were associated with seroreversion?

## Methods

### Study design and study population

The study design has been thoroughly described in former publications [19, 20]. In brief, this is a prospective longitudinal surveillance study in which adults were selected from the Belgian National Register of residents through multistage sampling. First, all reference persons of private households were divided into strata based on region of residence, gender and age group. Households were then randomly selected in each stratum, as primary sampling units. Within a selected household, all members aged 18 years and older were eligible for participation. The inclusion criterion of the study was having an official residence in Belgium at the moment of the sampling. Living in an institution (nursing homes, prisons, religious communities or cloisters, psychiatric institutions,…) or being under 18 years were exclusion criteria. A total of 1,339 adults belonging to 634 households were invited to participate in a pilot phase of the study. Sample size calculations indicated that 1,200 individuals in each of the 3 Belgian regions (thus a total of 3,600) would be large enough to obtain regional estimates on SARS-CoV-2 seroprevalence with sufficiently high precision [19]. Based on these predictions and on the participation scheme obtained during the pilot phase, another 12,862 individuals in 7,598 households were selected to supplement the baseline data collection point. For practical reasons, invitations to participate were sent out in 3 distinct time batches within a period of 4 weeks. Because the fieldwork procedures were not modified between the pilot and supplementary baseline data collection phases, the observations gathered in these two phases were merged into a total “wave 1” dataset. This allowed to increase the power of the analysis and to assess finetuned trends in antibody seroprevalence over an expanded timeline during the first study period. Finally, the wave 1 participants who agreed to follow-up were reinvited for the wave 2 and wave 3 data collection points. People were thus contacted 3 times for the study: between 25/03/2021 and 15/06/2021 for wave 1, between 23/09/2021 and 28/10/2021 for wave 2 and between 25/01/2022 and 08/02/2022 for wave 3. Participants got access to their test results through the project website or by phone, using a code.

### Data collection

Saliva samples were obtained from the participants through self-collection. People selected for the study received an invitation letter, two consent forms, an Oracol® tube (Malvern Medical Developments Ltd) for collecting saliva, a user’s guide (including an online video) on how to proceed and how to obtain their test result, a survey questionnaire (paper or online) and a prepaid return envelope. A third trusted party (Statbel, the national office of statistics) detained the participants’ names and addresses for sending out the follow-up invitations and survey material. The following topics were included in the questionnaires based on their potential association with COVID-19 antibody status: (1) sociodemographic information, (2) presence of chronic diseases, (3) occupational status, (4) financial situation, (5) access to health care services, (6) mental health, (7) social contacts, (8) lifestyle, (9) possible contact with SARS-CoV-2 virus and consequences, (10) adherence to policy measures, (11) vaccination status, and (12) attitude towards vaccination. A selection of relevant topics was considered for this study. Data were collected from 29th March 2021 until 25th April 2022.

### Assessment of outcome variables

The saliva samples were returned by post and analysed in Sciensano’s laboratories. Semi-quantitative measurements of anti-receptor-binding domains IgG (anti-RBD IgG) were performed using the WANTAI SARS-CoV-2 IgG ELISA (Quantitative Wantai Bio-Pharm, cat n° WS-1396) customized for saliva *(in house* protocol). The cut-off value for anti-RBD IgG positivity in saliva was previously established using PCR-confirmed samples from adults for whom both serum and saliva were available. Saliva from positive PCR cases were tested using the *in house* ELISA-protocol. Assay performance at each individual cut-off was evaluated using ROC (Receiver Operating Characteristic) analyses and a specificity-optimized cut-off value for anti-RBD IgG positivity in saliva was determined. This cut-off was used to create a binary variable reflecting the presence of SARS-CoV-2 antibodies in saliva (positive or negative test result) with a specificity of 96.7% and a sensitivity of 95.1%. The second outcome variable in our study is that of seroreversion, defined as passing from a positive to a negative antibody test result from one data collection point to the next.

### Assessment of potential determinants

The potential determinants of antibody seroposivity and/or seroreversion in this study comprised baseline measures of sociodemographic and occupational information (age, sex, region of residence, living situation, working in the health care sector), health and biological characteristics (self-rated health, activity limitations, blood type), important health risk factors (obesity and smoking) and chronic disease status. The latter expressed having at least one chronic disease or condition from a list of 12 that were defined by the Superior Health Council as priority criteria for COVID-19 vaccination in Belgium [21]. Information regarding COVID-19 illness and vaccination was gathered at each data collection point. Information on age, sex and region of residence was obtained directly from the national register. All other potential determinants were assessed through self-administered questionnaires. Validated instruments were used to measure mental and social constructs. Supplementary file 1 provides detailed information on all covariates comprised in the analyses, including the vaccination status and the presence of chronic diseases and conditions. People were categorized into three groups according to their vaccination status at each wave: unvaccinated people (i.e. not having received any COVID-19 vaccine at all); people partially vaccinated (i.e. having received just one dose of a double-dose COVID-19 vaccine, so not including unique-dose vaccines such as Johnson & Johnson), or having completed the basic vaccination less than 3 weeks ago; and people fully vaccinated (i.e. having received a complete vaccination scheme with or without a booster shot).

### Statistical analyses

First, we performed the trend analysis of community-wide SARS-CoV-2 seroprevalence using post-stratification survey weights. Time trends were assessed through logistical regressions with contrast statements using orthogonal polynomial coefficients and taking into account the survey settings. Weights were calculated with reference to both the Belgian population structure on January 1, 2021 established by Statbel and the official national vaccination records. Weighted seroprevalence estimates with 95% confidence intervals were calculated for seven time points during the full study period. Details on the weight calculations are provided in Supplementary file 2.

Subsequently, we assessed potential factors associated with the presence of SARS-CoV-2 antibodies in saliva samples. First, a database was constructed with all observations from the three data collection points. The association between potential determinants and the presence of SARS-CoV-2 antibodies was assessed at the level of the observations. Potential determinants included both fixed individual characteristics (like age and sex) and time varying characteristics (e.g. date of last vaccination). To take into account within-subject observations across the three data collection points, multilevel logistic regressions with three levels (observation, individual, household) were modelled, including the week number when the saliva was collected as covariate in the model. When the lowest unit of analysis was the observation (not the individual) no weights were used, but the factors that were used to calculate the survey weights (age group, sex and region) were included as co-variates in the models. The analyses were executed with the PROC GLIMMIX procedure in SAS® [22] and were conducted separately for fully vaccinated and unvaccinated people. Observations of the partially vaccinated were not involved in these analyses. A two-step approach was applied: first, the association of each independent variable with the SARS-CoV-2 antibody outcome was assessed separately. Next, the variables found to be significantly associated (*p* < 0.05) with having SARS-CoV-2 antibodies in the univariate analyses were modelled in a multivariable logistic regression with age and gender. If associations remained significant (*p* < 0.05), the interaction of the variable with time (week number) was also tested. This allowed assessing whether the associations changed over time.

Lastly, antibody seroreversion was examined among individuals with at least two consecutive observation points. Possible factors associated with seroreversion were additionally explored.

All analyses were conducted with SAS 9.4. A *p*-value < 0.05 was considered statistically significant.

## Results

### Study population

**Fig. 1:**
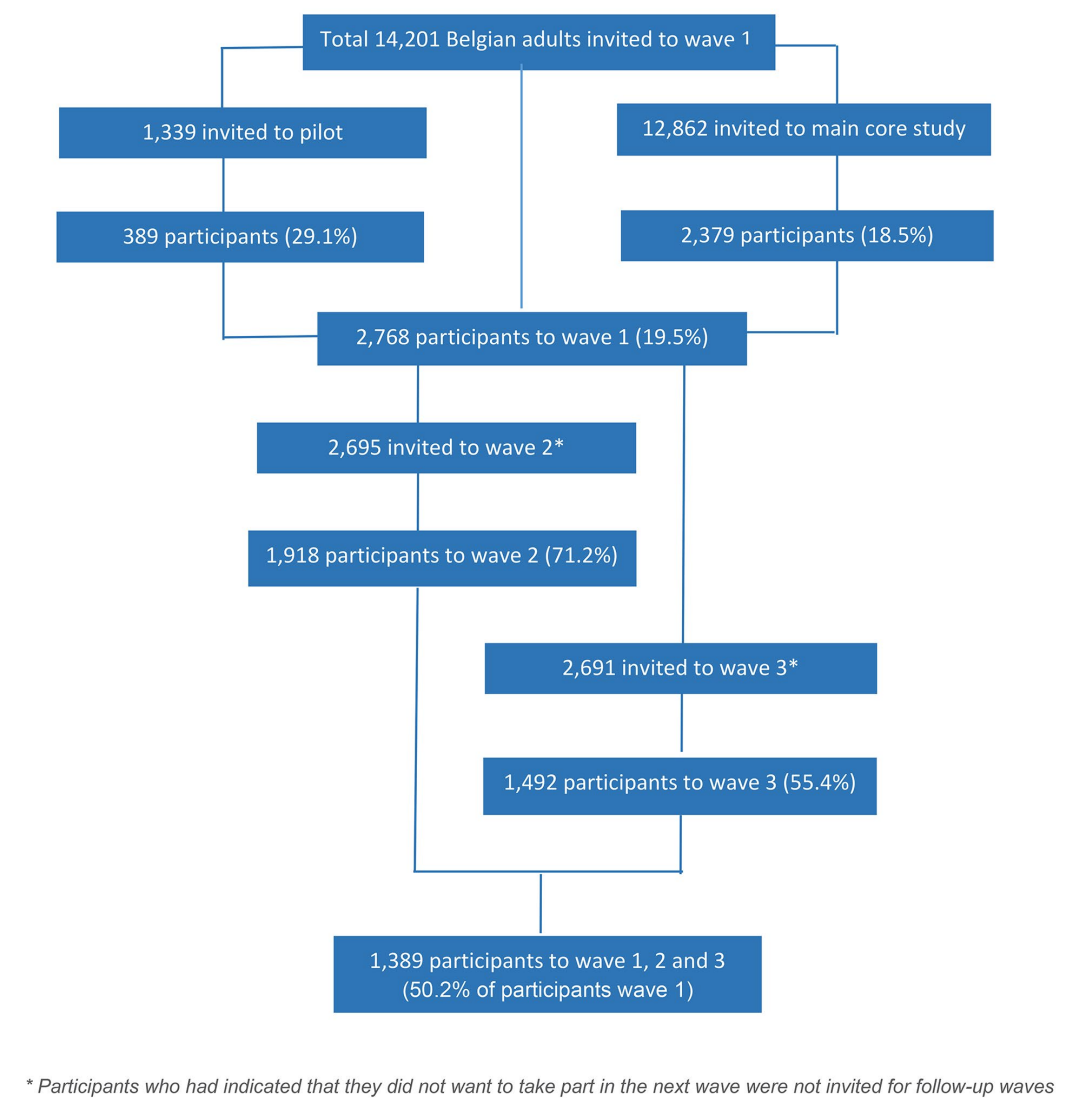
Participation flow among Belgian adults

Figure 1 indicates the participation flow across the different study waves. Out of 14,201 baseline invitations 2,768 individuals (19.5%) participated in wave 1, among whom 1,389 (50.2%) in all three waves. Merging the data from the three waves added up to 6,178 valid observations: 4,205 observations in individuals who were fully vaccinated at the time saliva was collected, 852 observations in those who were partially vaccinated and 1,121 observations in the unvaccinated. Supplementary file 3 provides information on the total number of saliva samples collected per week during the study period, the evolution of the number of COVID-19 cases during this period (showing four epidemic waves), the evolution of the national vaccination coverage, and the geographical spread of the study participants by municipality.

Table 1 provides a comparison of the distribution of the participants by gender, age group and region of residence with the equivalent distribution in the total Belgian population. As shown, participation drop-out between wave 1 and wave 3 was considerable, especially in the two youngest age groups (18–29 years and 30–49 years). However, considering the characteristics of those who participated in the three waves, all population groups remained well represented.

**Table 1:** Distribution of participants by gender, age group and region in relation to this distribution in the Belgian population, 2021

		SalivaHIS sample Participants wave 1		SalivaHIS sample Participants wave 1, 2 and 3		Belgian population 2021 (18+)*	
		*N*	%	*N*	%	*N*	%
Gender	Men	1,248	45.1	646	46.5	4,494,752	48.8
	Women	1,520	54.9	743	53.5	4,714,364	51.1
Age group	18–29 years	423	15.3	133	9.6	1,654,143	17.9
	30–49 years	862	31.1	355	25.6	3,001,662	32.6
	50–69 years	825	29.8	459	33.1	2,956,849	32.1
	70 + years	658	23.8	442	31.8	1,596,462	17.3
Region	Flanders	1,160	41.9	610	43.9	5,363075	58.2
	Brussels	819	29.6	404	29.1	944,417	10.3
	Wallonia	789	28.5	375	27.0	2,901,624	31.5
Total		2,768	100.0	1,389	100.0	9,209,116	100.0

* Source: Statbel

### Prevalence trend of SARS-CoV-2 antibodies

The trend in the prevalence of antibodies to SARS-CoV-2 observed in the general adult population and separately among the fully vaccinated and unvaccinated individuals is presented in Table 2. In the general population, the prevalence of antibodies increased from 25.1% in the first half of April 2021 to 92.3% in March 2022 (*p*-value for linear trend < 0.0001). Although the prevalence of SARS-CoV-2 antibodies among the fully vaccinated was high throughout the study period, it still rose as time progressed. During the pilot phase in March 2021, a relatively low antibody prevalence was found among the fully vaccinated (81.3%), but this result is based on the rather small number (*n* = 18) of fully vaccinated in our sample at that time, mostly consisting of older people, with a very short time lag between the latest vaccine and saliva collection. Later, between May 2021 and April 2022, the prevalence of SARS-CoV-2 antibodies among the fully vaccinated people increased from 92.4 to 99.3% (*p*-value for linear trend 0.0003).

**Table 2:** Prevalence trend of SARS-CoV-2 antibodies in the population aged 18 years and older in Belgium, global results and results by vaccination status, March 2021 until April 2022 ^§^

	Total population°		Fully vaccinated and last vaccination in past 3 months		Fully vaccinated and last vaccination ≥ 3 months ago		Unvaccinated	
Period	n/N*	%** (95% CI)	n/N*	%** (95% CI)	n/N*	%** (95% CI)	n/N*	%** (95% CI)
29/3–11/4/21 _W1_	72/284	25.1 (18.7–31.6)	14/18	81.3 (64.9–97.7)	0/0		38/223	16.6 (10.1–23.2)
17/5–13/6/21 _W1_	609/1111	47.8 (44.1–51.5)	298/324	92.5 (89.0-95.9)	25/27	91.3% (80.2–100.0)	70/410	17.5 (13.3–21.6)
14/6–11/7/21 _W1_	550/780	69.0 (63.3–74.6)	335/350	96.3 (94.0-98.7)	38/38	100.0 (-)	34/175	21.9 (10.1–33.7)
27/9–24/10/21 _W2_	764/841	84.5 (79.3–89.6)	219/227	98.0 (96.4–99.7)	517/559	93.6 (91.4–95.8)	7/33	16.3 (2.1–30.6)
25/10–21/11/21 _W2_	663/713	89.1 (84.9–93.2)	127/134	97.8 (95.1–100.0)	509/535	96.0 (94.2–97.8)	7/24	24.8 (6.1–43.6)
24/1–20/2/22 _W3_	1049/1076	91.3 (87.1–95.5)	766/770	99.8 (99.6–100.0)	235/237	99.0 (97.6–100.0)	10/30	17.8 (0.4–35.3)
21/2–20/3/22 _W3_	229/242	92.3 (86.8–97.9)	133/134	99.9 (99.6–100.0)	87/88	99.3 (97.8–100.0)	3/13	11.7 (2.3–21.1)

^§^ Observations from samples that were taken outside the 7 defined time periods are not considered here

° Including partially vaccinated people

* Number seropositive /total

** Weighted percentage

W1 wave1

W2 wave 2

W3 wave 3

Within each of the 7 time periods considered for the trend analysis, antibody prevalence was higher in fully vaccinated people having received their last shot in the past 3 months than in those having received it more than 3 months ago, but the difference between these two groups was only statistically significant (*p* = 0.005) within one time period (September 27 to October 24). Regarding the unvaccinated people, there was a clear increase in the prevalence of SARS-CoV-2 antibodies during the first data collection point of the study, rising from 16.6% at the time of the pilot study to 21.9% by the end of wave 1. During wave 2 and 3, the number of unvaccinated people was too low to estimate sufficiently precise antibody prevalence rates by time period among them.

### Characteristics associated with SARS-CoV-2 seropositivity

The potential determinants of seropositivity were explored separately for observations of the fully vaccinated and unvaccinated individuals at the time of saliva collection (Tables 3 and 4). The only factor significantly associated with higher seropositivity rates in both the fully vaccinated and unvaccinated was a history of COVID-19 infection. Among the fully vaccinated, additional factors significantly associated with a higher seropositivity rate were being younger (age group 18–39 years versus 65 + years), having a bachelor diploma (versus those with only secondary education and lower), living with others (versus living alone), having no chronic disease (versus at least one chronic disease), having had a vaccine in the last 3 months (versus vaccine more than 3 months ago) and having received the nucleic-acid vaccine or a combination (versus a viral-vectored vaccine only). Factors significantly associated with a higher seropositivity rate among the unvaccinated population were having non-O blood type (versus O blood type) and being non-smoker (versus smokers) (Tables 3 and 4).

**Table 3:** Results of simple and multiple multilevel logistic regression analyses on antibody seropositivity for the fully vaccinated population including the observations from the three waves^1^

	Adjusted OR (95% CI)	Adjusted OR (95% CI)	*p*-value time interaction
Age group			0.1707
18–39 years	Ref	Ref	
40–64 years	0.43 (0.23–0.82)*	0.66 (0.33–1.34)	
65 + years	0.24 (0.13–0.43)*	0.49 (0.25–0.96)*	
Gender			
Man	Ref	Ref	
Woman	1.65 (1.17–2.33)*	1.49 (0.99–2.23)	
Region			
Flanders	Ref		
Brussels	1.03 (0.68–1.57)		
Wallonia	1.04 (0.67–1.60)		
Education			0.8584
Secondary or lower	Ref	Ref	
Bachelor	2.11 (1.32–3.38)*	2.44 (1.40–4.22)*	
Master or higher	1.54 (1.02–2.33)*	1.43 (0.89–2.30)	
Living situation			0.1329
Alone	Ref	Ref	
With others	1.70 (1.14–2.55)*	1.76 (1.10–2.83)*	
Health care worker			
Yes	1.83 (0.87–3.86)		
No	Ref		
At least one chronic disease			0.0692
Yes	0.47 (0.33–0.68)*	0.59 (0.37–0.94)*	
No	Ref	Ref	
Self-perceived health			
Good to very good	Ref	Ref	
Fair, bad to very bad	1.78 (1.19–2.66)*	0.96 (0.54–1.71)	
Long term limitation (GALI^2^)			
Limited	0.48 (0.32–0.71)*	0.69 (0.39–1.23)	
Not limited	Ref	Ref	
Blood type			
O blood type	Ref		
Non-O blood type	1.02 (0.66–1.58)		
Obesity (BMI ≥ 30 kg/m²)			
Yes	0.67 (0.44–1.03)		
No	Ref		
Smoking			
Yes	0.65 (0.40–1.07)		
No	Ref		
COVID-19 infection			0.0927
Yes	10.14 (3.19–32.24)*	12.55 (2.96–53.16)*	
No	Ref	Ref	
Time since last vaccination dose^3^			0.6514
< 3 months	1.38 (099-1.92)	1.76 (1.09–2.85)*	
≥ 3 months ago	Ref	Ref	
Type of vaccine received			0.1249
Nucleic-acid or combination	8.44 (5.90-12.07)*	7.70 (5.07–11.69)*	
Only viral-vectored	Ref	Ref	

^1^Vaccination status assessed at the moment the saliva test was done

^2^Global Activity Limitation Indicator

^3^Potentially a booster vaccination

**Table 4:** Results of simple and multiple multilevel logistic regression analyses on antibody seropositivity for the unvaccinated population including the observations from the three waves^1^,

	Crude OR (95% CI)	Adjusted OR (95% CI)	*p*-value time interaction
Age group			
18–39 years	Ref	Ref	
40–64 years	1.06 (0.71–1.58)	0.81 (0.45–1.48)	
65 + years	0.86 (0.45–1.64)	0.55 (0.18–1.70)	
Gender			
Man	Ref	Ref	
Woman	1.34 (0.94–1.90)	0.98 (0.56–1.69)	
Region			
Flanders	Ref		
Brussels	1.44 (0.92–2.26)		
Wallonia	1.25 (0.79–1.97)		
Education			
Secondary or lower	Ref		
Bachelor	1.13 (0.73–1.77)		
Master or higher	0.96 (0.62–1.48)		
Living situation			
Alone	Ref		
With others	1.25 (0.71–2.20)		
Health care worker			
Yes	0.97 (0.41–2.28)		
No	Ref		
At least one chronic disease			
Yes	1.14 (0.69–1.88)		
No	Ref		
Self-perceived health			
Good to very good	Ref		
Fair, bad to very bad	1.43 (0.16–12.82)		
Long term limitation (GALI^2^)			
Limited	0.63 (0.31–1.28)		
Not limited	Ref		
Blood type			0.9803
O blood type	Ref	Ref	
Non-O blood type	1.67 (1.05–2.67)*	1.74 (1.01–3.01)*	
Obesity (BMI ≥ 30 kg/m²)			
Yes	1.12 (0.65–1.92)		
No	Ref		
Smoking			0.4346
Yes	0.43 (0.25–0.74)*	0.33 (0.13–0.82)*	
No	Ref	Ref	
Last COVID-19 infection^3^			0.0825
< 3 months ago﻿	14.23 (8.10–25.0)*	16.59 (7.10-38.77)*	
≥ 3 months ago	8.99 (4.83–16.71)*	7.61 (2.97–19.52)*	
No COVID-19 infection	Ref	Ref	

^1^Vaccination status assessed at the moment the saliva test was done

^2^Global Activity Limitation Indicator

^3^potentially a booster vaccination

The association between the chronic disease indicator and seropositivity among the fully vaccinated people was further explored in a specific analysis. For that, the chronic disease indicator was replaced by the 12 diseases and conditions separately. Detailed results are presented in Supplementary file 4. The main finding is that, after adjustment for the potential confounders, the fully vaccinated people with a neurological disease or with a transplant were significantly less inclined to present a seropositive test (respectively [ORa 0.33; 95% CI 0.13–0.84] and [ORa 0.01; 95% CI < 0.001–0.07]) compared to people without those problems.

### Seroreversion and associated characteristics

Seroreversion, defined here as passing from a positive to a negative antibody test from one data collection point to the next, was assessed between the first and second data collection points (waves 1 and 2) and between the second and third data collection points (wave 2 and 3). Out of 909 people with a positive antibody test in wave 1, 32 (3.5%) seroreversed in wave 2. Out of 1,065 seropositive people in wave 2, only 8 (0.8%) seroreversed in wave 3. So in total, seroreversion occurred in 40 cases during the study period. Seroreversion was significantly lower (0.3%) among the fully vaccinated people having received their latest vaccine in the last 3 months than among those who received their latest vaccine more than 3 months ago (2.7%) (OR 0.13; 95%CI 0.04–0.42). The percentage of seroreversion reached 36.0% in people who were not or partially vaccinated.

## Discussion

### Time trends

We aimed to determine the prevalence and evolution of SARS-CoV-2 antibodies in the general population aged 18 years and older during the 13-months study period. The increase in SARS-CoV-2 antibody prevalence, from 25.1% in April 2021 to 92.3% in March 2022, was the consequence of the vaccination campaign during this period. These rates were found to be consistently lower in comparison to the seroprevalence rates from the Belgian blood donors study [2] between April and December 2021. It is unlikely that salivary test we used explains this difference, as it was validated against COVID-19 PCR and paired serum/saliva samples with 95.1% sensitivity. Rather, our results highlight the value of general population studies to complement the scope of national serological surveillance.

Importantly, this study allowed assessing the trend in prevalence of SARS-CoV-2 antibodies in both vaccinated and unvaccinated people from the general population. In vaccinated people, the seroprevalence was high throughout the study period, but increased slightly as time progressed. However, a seroprevalence of 81.3% should be interpreted with caution due to the very small number of vaccinated participants in the pilot phase period. From the next period onwards, the number of vaccinated participants was high enough to give reasonably precise estimates. Here, the prevalence of SARS-CoV-2 antibodies in fully vaccinated people increased from 92.5 to 99.9% between May 2021 and March 2022. The lower rates found in the beginning probably result from the vaccine campaign starting with older people and people with chronic morbidities, who may have a lower immune response to vaccination. The increase in immune response over time is possibly due to multiple exposures to the antigen as time progressed (through COVID-19 infection or vaccine, including booster). Among the unvaccinated people, the prevalence of SARS-CoV-2 antibodies increased during wave 1, but was variable across time in wave 2 and wave 3, and lower than expected. Again, the low number (*N* = 100) of unvaccinated people in these periods call for caution in interpreting the results. The low antibody prevalence among unvaccinated people could derive from them adhering more strickly to the sanitary measures to prevent infection(e.g. lock-down, tele-working, mask wearing). Indeed, the proportion of participants who reported a history of a COVID-19 infection was lower than expected from the official COVID-19 infection statistics.

### Characteristics associated with SARS-CoV-2 seropositivity

Among fully vaccinated people, seropositivity was significantly lower in those with a chronic disease, more particularly a neurological disease or a transplant. This result needs further investigation, as our study relies on self-report and a limited number of affected individuals. However, other findings support our results. For instance, patients with multiple sclerosis (a neurological affection), receiving disease-modifying therapies showed a reduced humoral immunity after SARS-CoV-2 vaccination [23].In addition, the seropositivity results in our group of participants with a transplant (58.3%) were remarkably similar to the SARS-CoV-2 anti-Spike seroprevalence of 52.4% found in a study among renal transplant patients [24, 25].

Social factors were also influencial. First, antibodies were more often present in people with a bachelor diploma who had higher seropositivity rates compared to those with a lower education. A possible explanation is that higher socio-economic status is associated with a better health status and behaviors, hence a stronger immune system. However, this was not confirmed among people with a master degree and above. Second, vaccinated people living with others had higher seropositivity rates compared to those living alone. Possibly they have a greater chance to be exposed to the virus. Studies have shown that living with children for instance increases the risk of SARS-CoV-2 infection [26, 27].

Finally, although both types of anti-COVID vaccines (nucleic-acid and viral-vectored) have demonstrated their effectiveness and their association with antibody development, some studies showed a higher seroprevalence among people who received a nucleic-acid vaccine compared to those with a viral-vectored vaccine [28]. This was also observed in our study. Seroprevalence was lower in the virus-vectored vaccine group compared to those having a nucleic-acid vaccine, whether delivered in a basic vaccination scheme or as a booster.

Among the unvaccinated people, seropositivity rate was lower in those with a O blood type compared to in those with an non-O blood type. A systematic review and meta-analysis indicated that blood group A may be a risk factor for COVID-19, whereas blood group O appears to be somewhat protective [29]. To what extent and how this relates with our findings remains unclear.

### Seroreversion

Seroreversion occurred in only 40 study participants. This low number may be related to the surge of Delta and Omicron variants of the virus between wave 2 and 3, resulting in many reinfections, hence few seroreversions.

Clearly, the time since the latest vaccination was an important predictor of seroreversion. Seroreversion was also much higher among the partially or unvaccinated people compared to people who were fully vaccinated. This confirms findings that antibodies developed following vaccination or following a mix of vaccination and COVID-19 infection were more robust and waned less rapidly than those developed after natural infection only [30, 31].

### Limitations and strengths of the study

Our study has some important limitations regarding serological surveillance. First, we opted to detect antibodies in saliva, while serum-based methods are the preferred reference for seroprevalence studies. Still, our in-house SARS-CoV-2 RBD IgG ELISA test on saliva had shown high sensitivity and specificity. Unfortunately it made no distinction between antibodies from natural infection and from vaccination. Furthermore, the outcome reported in this study was dichotomous (presence or absence of antibodies), which had an impact on the level of analyses (less precision), but also on the interest of the study to participants. Indeed, providing the test result to the participants was initially an important incentive, but their motivation for follow-up decreased as the level of protection was unknown, the vaccine roll-out was fast and the epidemic was on a decline. Finally, the saliva collection was executed by the participants themselves, without supervision. The method to collect saliva (Oracol®) is designed for self-use and much effort had been put in giving clear instructions in a leaflet and a video. Nevertheless, it appeared that 17% of the initial swap samples down to 9% of the wave 3 swap samples did not contain enough saliva to be analysed.

The study also bears important strengths. It is a population-based probability sample including residents from 317 of the 581 Belgian municipalities. Even though non-response and drop-out were substantial and biases are inevitable, the use of post-stratification weights with both the national register and the exhaustive Belgian vaccination record database as auxiliary data sets, ensured that results were as representative as possible of the Belgian population aged 18 years and above.

Furthermore, the questionnaires that accompanied the three data collection waves allowed to gather extensive information from the participants in many different domains: socio-demographic information, health related factors, health behaviors, COVID-19 infection, vaccination status, etc. Additionally, over 90% of the study participants agreed that their saliva samples could be stored in a biobank and that their results could be linked with administrative databases for further research.

## Conclusions

This study provided useful information for monitoring the COVID-19 pandemic in the general adult population in Belgium. It allowed to identify, among vaccinated and unvaccinated people, factors that were associated with a lower humoral immune response and provided some insights on waning of SARS-CoV-2 antibodies among vaccinated people in the general population.

Because COVID-19 and other viruses continue to be a public health concern, monitoring antibodies at population level remains useful, as is done in other countries [32, 33], but improvements in this type of study set-up are necessary:

- The methods used to test seropositivity should be able to distinguish between antibodies generated as a result of vaccination and antibodies generated as a result of infection.
- Future monitoring procedures could consider postal collection of blood samples by means of a finger prick for example. However, this may have an impact on participation bias and rate, since a blood collection is more invasive than a saliva collection.
- Growing evidence is available on the antibody levels associated with protection against infection, also for SARS-CoV-2 [34]. A surveillance system should be able to assess the level of protection of the general population, in specific population groups and for different variants.
- From a cost-effectiveness point of view, it should be investigated if such a surveillance could be integrated in a more global serosurveillance system in which antibodies against various pathogens are included, as is for instance the case in the Netherlands, where monitoring of the SARS-CoV-2 seroprevalence has been integrated in the national seroepidemiological (PIENTER) studies [35, 36].

### Electronic supplementary material

Below is the link to the electronic supplementary material.

Supplementary Material 1

Supplementary Material 2

Supplementary Material 3

Supplementary Material 4

## Data Availability

Data are available on reasonable request. The statistical codes that support the findings of this study are available from the corresponding author on reasonable request.

## Abbreviations

SARS-CoV-2: Severe acute respiratory syndrome coronavirus 2
COVID-19: Coronavirus Disease 2019
IgG: Imunnoglobuline G
ELISA: Enzyme-linked immunoassay
RBD: Receptor-binding domain
PCR: Polymerase chain reaction
ROC: Receiving operating characteristic
SAS: Statistical analysis software
PIENTER: Peiling Immunisatie Effect Nederland Ter Evaluatie van het Rijksvaccinatieprogramma

## References

[1] Repatriated Belgian tests positive for coronavirus. Reuters [Internet]. 2020 Feb 4 [cited 2022 Dec 5]; https://www.reuters.com/article/us-china-health-belgium-idUSKBN1ZY0OK.

[2] Epistat. – COVID-19 Monitoring [Internet]. [cited 2020 Dec 13]. https://epistat.sciensano.be/covid/.

[3] World Health Organisation. Population-based age-stratified seroepidemiological investigation protocol for COVID-19 virus infection [Internet]. 2020. https://apps.who.int/iris/bitstream/handle/10665/331656/WHO-2019-nCoV-Seroepidemiology-2020.1-eng.pdf?sequence=1&isAllowed=y

[4] Figueiredo-Campos P, Blankenhaus B, Mota C, Gomes A, Serrano M, Ariotti S (2020). Seroprevalence of anti‐SARS‐CoV‐2 antibodies in COVID‐19 patients and healthy volunteers up to 6 months post disease onset. Eur J Immunol.

[5] Iyer AS, Jones FK, Nodoushani A, Kelly M, Becker M, Slater D (2020). Persistence and decay of human antibody responses to the receptor binding domain of SARS-CoV-2 spike protein in COVID-19 patients. Sci Immunol.

[6] Rostami A, Sepidarkish M, Fazlzadeh A, Mokdad AH, Sattarnezhad A, Esfandyari S (2021). Update on SARS-CoV-2 seroprevalence: regional and worldwide. Clin Microbiol Infect.

[7] Neuhauser H, Rosario AS, Butschalowsky H, Haller S, Hoebel J, Michel J (2022). Nationally representative results on SARS-CoV-2 seroprevalence and testing in Germany at the end of 2020. Sci Rep.

[8] Lippi G, Henry BM, Plebani M. Anti-SARS-CoV-2 antibodies testing in recipients of COVID-19 vaccination: why, when, and how? Diagnostics (Basel). 2021;11(6):941. 10.3390/diagnostics11060941 PMC8228868 34070341

[9] Herzog SA, De Bie J, Abrams S, Wouters I, Ekinci E, Patteet L (2022). Seroprevalence of IgG antibodies against SARS-CoV-2 - a serial prospective cross-sectional nationwide study of residual samples, Belgium, March to October 2020. Euro Surveill.

[10] Mortgat L, Verdonck K, Hutse V, Thomas I, Barbezange C, Heyndrickx L (2021). Prevalence and incidence of anti-SARS-CoV-2 antibodies among healthcare workers in Belgian hospitals before vaccination: a prospective cohort study. BMJ Open.

[11] Adriaenssens N, Scholtes B, Bruyndonckx R, Verbakel JY, De Sutter A, Heytens S (2022). Prevalence and incidence of antibodies against SARS-CoV-2 among primary healthcare providers in Belgium during 1 year of the COVID-19 epidemic: prospective cohort study protocol. BMJ Open.

[12] Boey L, Roelants M, Merckx J, Hens N, Desombere I, Duysburgh E (2022). Age-dependent seroprevalence of SARS-CoV-2 antibodies in school-aged children from areas with low and high community transmission. Eur J Pediatr.

[13] Meyers E, De Rop L, Deschepper E, Duysburgh E, De Burghgraeve T, Van Ngoc P et al. Prevalence of SARS-CoV-2 antibodies among Belgian nursing home residents and staff during the primary COVID-19 vaccination campaign. Eur J Gen Pract. 2022;1–9. 10.1080/13814788.2022.2149732 PMC10249443 36440533

[14] Pannus P, Neven KY, De Craeye S, Heyndrickx L, Vande Kerckhove S, Georges D (2022). Poor antibody response to BioNTech/Pfizer Coronavirus Disease 2019 vaccination in severe Acute Respiratory Syndrome Coronavirus 2-Naive residents of nursing homes. Clin Infect Dis.

[15] Grant R, Dub T, Andrianou X, Nohynek H, Wilder-Smith A, Pezzotti P (2021). SARS-CoV-2 population-based seroprevalence studies in Europe: a scoping review. BMJ Open.

[16] Randad PR, Pisanic N, Kruczynski K, Manabe YC, Thomas D, Pekosz A et al. COVID-19 serology at population scale: SARS-CoV-2-specific antibody responses in saliva. medRxiv [Internet]. 2020 May 26 [cited 2020 Aug 13]; https://www.ncbi.nlm.nih.gov/pmc/articles/PMC7273305/. 10.1128/JCM.02204-20 PMC7771435 33067270

[17] Vandermeulen C, Duysburgh E, Desombere I, Boey L, Roelants M. Seroprevalence of Sars-CoV-2 antibodies in school aged children in two regions with difference of COVID-19 disease. Validation study of saliva test for SARS-Cov-2 antibodies in children. Interim report [Internet]., KULeuven S. p. 28. https://www.sciensano.be/sites/default/files/limburg-validation-sars-cov2_report_20201112_final.pdf.

[18] Isho B, Abe KT, Zuo M, Jamal AJ, Rathod B, Wang JH (2020). Persistence of serum and saliva antibody responses to SARS-CoV-2 spike antigens in COVID-19 patients. Sci Immunol.

[19] Leclercq V, Van den Houte N, Gisle L, Roukaerts I, Barbezange C, Desombere I (2022). Prevalence of Anti-SARS-CoV-2 antibodies and potential determinants among the Belgian Adult Population: Baseline results of a prospective cohort study. Viruses.

[20] Leclercq V, Van der Heyden J. Prevalence of antibodies against the coronavirus (SARS-COV-2) in the population in Belgium. a prospective interventional study: the SalivaHIS cohort. 2021 Mar 11 [cited 2021 Oct 7]; https://osf.io/5tf8s/.

[21] Report. 9618 - Prioritization of at-risk groups for SARS-CoV-2 Vaccination (Phase 1b) [Internet]. FPS Public Health. 2021 [cited 2023 Mar 8]. https://www.health.belgium.be/en/report-9618-prioritization-risk-groups-sars-cov-2-vaccination-phase-1b.

[22] SAS® 9 (2013). 4 statements: reference.

[23] Holroyd KB, Healy BC, Conway S, Houtchens M, Bakshi R, Bhattacharyya S (2022). Humoral response to COVID-19 vaccination in MS patients on disease modifying therapy: Immune profiles and clinical outcomes. Mult Scler Relat Disord.

[24] Russo G, Lai Q, Poli L, Perrone MP, Gaeta A, Rossi M (2022). SARS-COV-2 vaccination with BNT162B2 in renal transplant patients: risk factors for impaired response and immunological implications. Clin Transpl.

[25] Kemlin D, Lemy A, Pannus P, Desombere I, Gemander N, Goossens ME (2022). Hybrid immunity to SARS-CoV-2 in kidney transplant recipients and hemodialysis patients. Am J Transpl.

[26] Lessler J, Grabowski MK, Grantz KH, Badillo-Goicoechea E, Metcalf CJE, Lupton-Smith C (2021). Household COVID-19 risk and in-person schooling. Science.

[27] Forbes H, Morton CE, Bacon S, McDonald HI, Minassian C, Brown JP (2021). Association between living with children and outcomes from covid-19: OpenSAFELY cohort study of 12 million adults in England. BMJ.

[28] Self WH. Comparative Effectiveness of Moderna, Pfizer-BioNTech, and Janssen (Johnson & Johnson) Vaccines in Preventing COVID-19 Hospitalizations Among Adults Without Immunocompromising Conditions — United States, March–August 2021. MMWR Morb Mortal Wkly Rep [Internet]. 2021 [cited 2022 Sep 6];70. https://www.cdc.gov/mmwr/volumes/70/wr/mm7038e1.htm. 10.15585/mmwr.mm7038e1 PMC8459899 34555004

[29] Liu N, Zhang T, Ma L, Zhang H, Wang H, Wei W (2021). The impact of ABO blood group on COVID-19 infection risk and mortality: a systematic review and meta-analysis. Blood Rev.

[30] Braeye T, Catteau L, Brondeel R, van Loenhout JAF, Proesmans K, Cornelissen L (2022). Vaccine effectiveness against onward transmission of SARS-CoV2-infection by variant of concern and time since vaccination, Belgian contact tracing, 2021. Vaccine.

[31] Cromer D, Juno JA, Khoury D, Reynaldi A, Wheatley AK, Kent SJ (2021). Prospects for durable immune control of SARS-CoV-2 and prevention of reinfection. Nat Rev Immunol.

[32] PIENTER Corona Study | RIVM [Internet]. [cited 2023 Mar 8]. https://www.rivm.nl/en/pienter-corona-study.

[33] Coronavirus (COVID-19). - Office for National Statistics [Internet]. [cited 2022 Jan 19]. https://www.ons.gov.uk/peoplepopulationandcommunity/healthandsocialcare/conditionsanddiseases.

[34] Feng S, Phillips DJ, White T, Sayal H, Aley PK, Bibi S (2021). Correlates of protection against symptomatic and asymptomatic SARS-CoV-2 infection. Nat Med.

[35] Verberk JDM, Vos RA, Mollema L, van Vliet J, van Weert JWM, de Melker HE (2019). Third national biobank for population-based seroprevalence studies in the Netherlands, including the Caribbean Netherlands. BMC Infect Dis.

[36] Vos ERA, den Hartog G, Schepp RM, Kaaijk P, van Vliet J, Helm K (2021). Nationwide seroprevalence of SARS-CoV-2 and identification of risk factors in the general population of the Netherlands during the first epidemic wave. J Epidemiol Community Health.

