# Supplementary material for: Prevalence of SARS-CoV-2 antibodies and associated factors in the adult population of Belgium: a general population cohort study between March 2021 and April 2022
Source: Arch Public Health. 2024 May 15;82:72. doi: 10.1186/s13690-024-01298-1 (PMC11094959; doi:10.1186/s13690-024-01298-1)
Supplement: Supplementary file 1 — Supplementary Material 1 [file 13690_2024_1298_MOESM1_ESM.docx]

**Supplementary file 1: Potential determinants considered in the analyses and way in which they were constructed**

| **Sociodemographic** **variables** | |
| --- | --- |
| Age group | Age was trichomized into the following age groups: 18-39 years, 40-64 years and 65+ years |
| Sex | Sex was based on the official information from the National Register |
| Region | Belgium is divided in 3 geographical areas: Flanders, Wallonia and the Brussels Capital Region |
| Education | Three categories were considered based on the highest diploma people achieved: a diploma of secondary education or lower, a bachelor degree and a master degree or higher |
| Living situation | Alone versus living with others |
| Health care worker | People where identified via the question “Do you work in the health care sector (institutional care, ambulatory care, home care)?” |
| **Health and biological characteristics** | |
| At least one chronic disease | Suffering from at least one of the following diseases or conditions: asthma, chronic bronchitis, high blood pressure, cardiovascular disease, diabetes, neurological disease, kidney disease, chronic liver disease, cancer (not blood cancer), blood cancer, condition affecting immune system (except HIV) or being a transplant patient. |
| Self-rated health (SRH) | SRH is based on the question “How is your health in general? Is it very good, good, fair, poor, or very poor?”(1). A dichotomic indicator was created: “Very good to good” versus “fair to very poor”. |
| Long term limitation | The Global activity limitation indicator (GALI) (1) was used. It is based on the question “For the past 6 months or more have you been limited in activities people usually do because of health problems?” The answer categories were dichotomized into “Moderate to severe limitations” and “No limitations”. |
| Blood type | Based on evidence of the impact of ABO blood group on COVID-19 infection risk and mortality (2) two categories were distinguished: O blood type and Non-O blood type |
| **Important health risk factors** | |
| Daily smoking | Smoking status was assessed with the question “Do you currently smoke? Yes, every day – Yes, occasionally – No, not at all” with the first category as outcome of interest. |
| Obesity | Obesity was defined as a BMI > 30 kg/m² based on self-reported weight and height |

| **COVID-19 illness and vaccination history** | |
| --- | --- |
| Last COVID-19 infection | Three categories were distinguished:   1. Last COVID-19 infection less than 3 months ago 2. Last COVID-19 infection 3 months ago or more 3. No known COVID-19 infection |
| Hospitalisation due to COVID-19 | This was a binary variable without further information on the time of the hospitalisation |
| COVID-19 vaccination status | Four categories were distinguished:   1. Fully vaccinated (i.e. having received at least a complete basic vaccination) with the last dose (possibly a booster) less than 3 months ago 2. Fully vaccinated (i.e. having received at least a complete basic vaccination) with the last dose (possibly a booster) more than 3 months ago 3. Partially vaccinated, i.e. having received at least one vaccination dose (other than Johnsson&Johnsson), but not a complete vaccination scheme 4. Not vaccinated, i.e. not having received any vaccination at all |
| Type of vaccine received | This indicator was only considered for fully vaccinated people. Two categories were considered: those having received at least one dose of a nucleic-acid vaccine (Pfizer or Moderna), possibly as a booster versus those having received only a viral-vectored vaccine (Johnsson&Johnsson or AstraZeneca) |

1. Cox B, Oyen HV, Cambois E, Jagger C, Roy S le, Robine JM, et al. The reliability of the Minimum European Health Module. Int J Public Health. 2009 Apr 1;54(2):55–60.

2. Liu N, Zhang T, Ma L, Zhang H, Wang H, Wei W, et al. The impact of ABO blood group on COVID-19 infection risk and mortality: A systematic review and meta-analysis. Blood Reviews. 2021 Jul 1;48:100785.
