# Supplementary material for: Prevalence of SARS-CoV-2 antibodies and associated factors in the adult population of Belgium: a general population cohort study between March 2021 and April 2022
Source: Arch Public Health. 2024 May 15;82:72. doi: 10.1186/s13690-024-01298-1 (PMC11094959; doi:10.1186/s13690-024-01298-1)
Supplement: Supplementary file 2 — Supplementary Material 2 [file 13690_2024_1298_MOESM2_ESM.docx]

**Supplementary file 2: Calculation of weights**

The principle behind estimating population parameters using a probability sample is that each person in the sample represents, besides him-/herself, an entire slice of the population. For example, in a simple random sample of 1% of a population of 100,000 individuals, each person in the sample represents 1,000 persons in the population. In this case, each person will be attributed a weight of 1,000.

The weight for each sampled individual in this study is the reciprocal of a post stratification factor for each combination of region, age group and gender. Strata which are underrepresented in the sample will receive a higher weight, and those that are overrepresented a lower one. E.g. because of the relative oversampling of the Brussels region, the average weight for participants living in Brussels was 4 times lower than the average weight of participants from Flanders. The application of weights cannot exclude selection bias, but ensures that the weighted distribution of the sample by age group, gender and region matches exactly these distribution in the Belgian population.

To study how the seroprevalence evolved over the study period, we defined 7 time periods in which sufficient data were collected to calculate reasonably precise estimates (Table A2.1). For each of these time periods, the participants were considered as a sample on itself and weights were calculated separately for each of these samples. Data collected outside the defined time periods were not taken into consideration in the trend analysis.

Table A2.1 Periods considered for the time trend analyses, SalivaHIS study, Belgium 2021-2022

| Period | Wave | Week number | Dates | Number of participants |
| --- | --- | --- | --- | --- |
| 1 | 1 (pilot) | 13-14 | 29/3-11/4/2021 | 284 |
| 2 | 1 | 20-23 | 17/5-13/6/2021 | 1,111 |
| 3 | 1 | 24-27 | 14/6-11/7/2021 | 780 |
| 4 | 2 | 39-42 | 27/9-24/10/2021 | 841 |
| 5 | 2 | 43-47 | 25/10-21/11/2021 | 713 |
| 6 | 3 | 4-7 | 24/1/-20/2/2022 | 1,076 |
| 7 | 3 | 8-11 | 21/2/-20/3/2022 | 242 |

During the data collection period, the vaccination campaign in Belgium was running at full speed. On 29/03/2021, when the pilot study began, 11.3% of the population had received their first vaccination dose. On 25/04/2022, when the last saliva sample was collected, the percentage of primo-vaccinated had increased to 79.2%. This of course had a major impact on the evolution of the prevalence of SARS-CoV-2 antibodies among the population during the SalivaHIS study period. When comparing the vaccination rates in our sample with the official vaccination coverages in the population, it appeared that there were important differences, especially in the Brussels Capital region. The most plausible hypothesis is that this was the result of a difference in participation rate in the study between vaccinated and unvaccinated people. To reduce the bias in the estimates because of this differential participation rate, weights to assess the trends in the prevalence of SARS-CoV-2 antibodies did not only take into account regional, age and gender differences between the sample and the general population, but also differences in the vaccination status ratio. This was done by multiplying the initial weight with a correction factor. The correction factor was obtained by dividing, per region, the number of people by vaccination status in the population by the corresponding number of people in the sample. In this way the weighted sample distribution by vaccination status and region matched this distribution in the general population.
