# Supplementary material for: Prevalence of SARS-CoV-2 antibodies and associated factors in the adult population of Belgium: a general population cohort study between March 2021 and April 2022
Source: Arch Public Health. 2024 May 15;82:72. doi: 10.1186/s13690-024-01298-1 (PMC11094959; doi:10.1186/s13690-024-01298-1)

**Supplementary file 3: Saliva sample collection during the study period, evolution of vaccination coverage during the study period and geographical distribution of the sample**

Figure A3.1 Number of saliva samples collected per week*


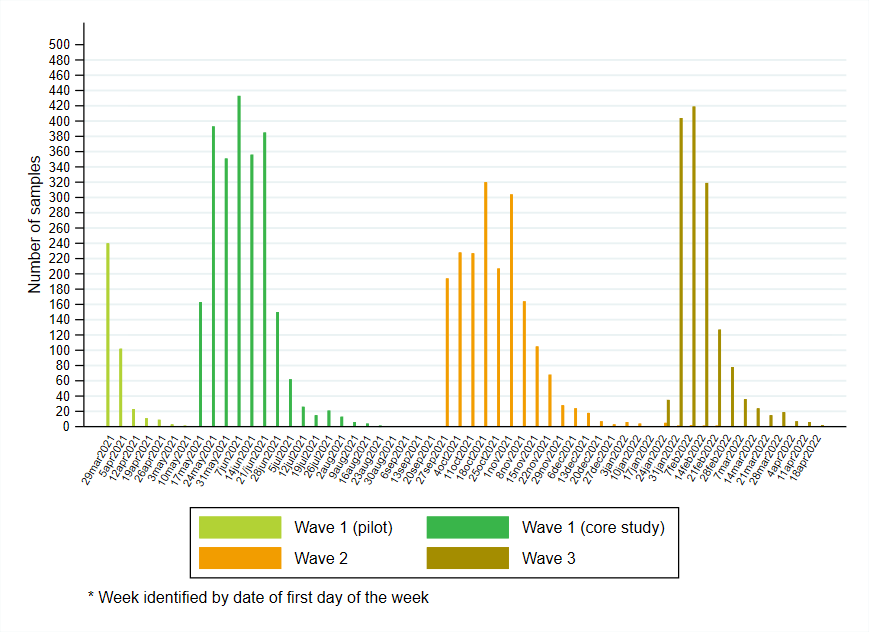


Figure A3.2 Evolution of vaccination coverage (% of total population) and weekly number of new COVID-19 infections in Belgium during the SalivaHIS study period

**
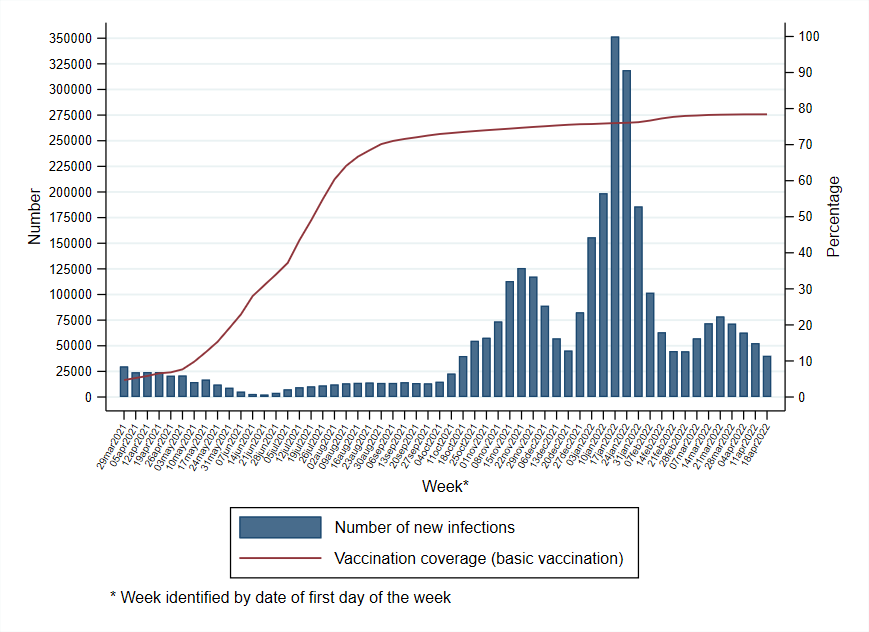
**=

Figure A3.3 Belgian municipalities with at least one participant to the 3 waves of the study period


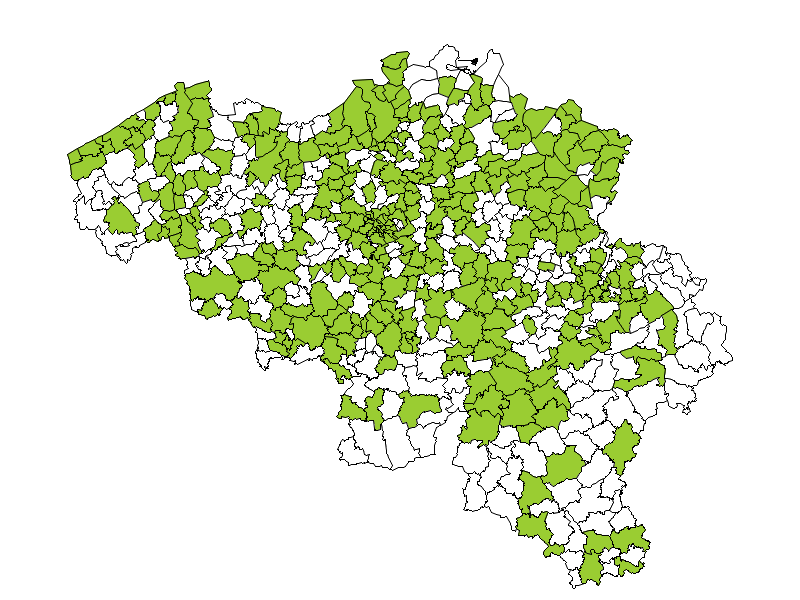

Supplement: Supplementary file 3 — Supplementary Material 3 [file 13690_2024_1298_MOESM3_ESM.docx]
