# Supplementary material for: Prevalence of SARS-CoV-2 antibodies and associated factors in the adult population of Belgium: a general population cohort study between March 2021 and April 2022
Source: Arch Public Health. 2024 May 15;82:72. doi: 10.1186/s13690-024-01298-1 (PMC11094959; doi:10.1186/s13690-024-01298-1)
Supplement: Supplementary file 4 — Supplementary Material 4 [file 13690_2024_1298_MOESM4_ESM.docx]

Supplementary file 4: Exploration of presence of SARS-CoV-2 antibodies in relation to specific chronic disease status in the vaccinated population

Table A4.1 Percentage of positive SARS-CoV-2 antibody observations by group of diseases reported among the fully vaccinated population

|  | Seropositivity | | N No of participants with such disease |
| --- | --- | --- | --- |
|  | n / N* | % (95% CI) |  |
| Asthma | 238 / 248 | 96.0 (93.5-98.4) | 185 |
| Chronic bronchitis | 105 / 114 | 92.1 (87.2-97.1) | 88 |
| High blood pressure | 722 / 756 | 95.5 (94.0-97.0) | 503 |
| Cardiovascular disease | 215 / 229 | 93.9 (90.8-97.0) | 156 |
| Diabetes | 196 / 213 | 92.0 (88.4-95.7) | 163 |
| Neurological disease | 94 / 106 | 88.7 (82.6-94.7) | 87 |
| Kidney disease | 39 / 42 | 92.9 (85.1-100.0) | 37 |
| Chronic liver disease | 25 / 26 | 96.2 (88.8-100.0) | 22 |
| Cancer (not blood cancer) | 104 / 112 | 92.9 (88.1-97.6) | 70 |
| Blood cancer | 27 / 31 | 87.1 (75.3-98.9) | 19 |
| Condition affecting immune system (except HIV) | 82 / 85 | 96.0 (95.3-96.6) | 57 |
| Being a transplant patient | 7 / 12 | 58.3 (30.4-86.2) | 8 |

*****Number of observations with a positive test result / total observations within fully vaccinated population

Table A4.2 Association between seropositivity and presence of specific chronic diseases. Results of the univariate and multivariate regression analyses for observations of the fully vaccinated

|  | Crude OR  (95% CI) | Adjusted OR°  (95% CI) |
| --- | --- | --- |
| Asthma  Chronic bronchitis  High blood pressure  Cardiovascular disease  Diabetes  Neurological disease  Kidney disease  Chronic liver disease  Cancer (not blood cancer)  Blood cancer  Condition affecting immune system (except HIV)  Transplantation | 1.04 (0.52-2.10)  0.50 (0.23-1.09)  0.93 (0.61-1.42)  0.62 (0.33-1.15)  0.52 (0.29-0.93)*  0.31 (0.15-0.64)*  0.57 (0.15-2.07)  1.07 (0.13-8.80)  0.53 (0.24-1.21)  0.26 (0.08-0.90)*  1.04 (0.31-3.52)  0.05 (0.01-0.22)*** | 0.91 (0.40-2.05)  1.05 (0.39-2.85)  1.48 (0.87-2.53)  0.60 (0.29-1.27)  0.69 (0.34-1.41)  0.33 (0.13-0.84)*  0.34 (0.07-1.60)  2.43 (0.15-40.32)  0.81 (0.30-2.21)  1.80 (0.13-25.40)  4.26 (0.39-46.76)  0.01 (<0.001-0.07)*** |

° adjusted for age group, gender, education, living situation, self-perceived health, long term limitations, history of COVID-19 infection, time since last vaccination dose and type of vaccine received

* p < 0.05

*** p < 0.001
